# How to implement a radiologist led whole-body MRI screening program

**DOI:** 10.1093/radadv/umag014

**Published:** 2026-03-10

**Authors:** Andrea S Kierans, Keith D Hentel, Katerina Dodelzon, George Shih, Martin R Prince, Joshua Lantos, Melissa K Frey, Ravi N Sharaf, Robert J Min, Preethi Guniganti

**Affiliations:** Weill Cornell Medical College, Department of Radiology, New York City, NY, USA; Weill Cornell Medical College, Department of Radiology, New York City, NY, USA; Weill Cornell Medical College, Department of Radiology, New York City, NY, USA; Weill Cornell Medical College, Department of Radiology, New York City, NY, USA; Weill Cornell Medical College, Department of Radiology, New York City, NY, USA; Weill Cornell Medical College, Department of Radiology, New York City, NY, USA; Weill Cornell Medical College, Division of Gynecologic Oncology, Department of Obstetrics and Gynecology, New York City, NY, USA; Weill Cornell Medical College, Division of Gastroenterology, Department of Healthcare Policy and Research, New York City, NY, USA; Weill Cornell Medical College, Department of Radiology, New York City, NY, USA; Weill Cornell Medical College, Department of Radiology, New York City, NY, USA

**Keywords:** screening whole-body MRI, cancer screening, preventative imaging

## Abstract

Screening whole-body MRI (WB-MRI) is gaining increasing attention as a tool for early disease detection, with growing adoption driven largely by consumer demand and direct-to-consumer private platforms. While WB-MRI has demonstrated utility in high-risk populations, its use in asymptomatic individuals remains controversial due to concerns about low diagnostic yield, false positives, overdiagnosis, and the lack of survival outcome data. Despite these limitations, the popularity of WB-MRI is expected to rise given the aging population and aggressive marketing by direct-to-consumer companies, underscoring the need for thoughtful and proactive engagement by radiologists. Radiologists have an obligation to ensure that scientific rigor, ethical oversight, and multidisciplinary collaboration guide the expansion of WB-MRI. This review outlines the current evidence and evolving landscape of screening WB-MRI, describes the development and implementation of a program within an academic radiology practice, and discusses the downstream implications and cost-effectiveness of WB-MRI screening. As this technology continues to expand beyond traditional indications, radiologists must play a leading role in defining best practices and ensuring that implementation remains evidence-based, transparent, and patient-centered.

## Teaser

Summary statement

This review evaluates the current evidence, practical implementation, and ethical and economic considerations regarding screening whole-body MRI in asymptomatic individuals, emphasizing radiologists’ essential role in guiding its evidence-based, patient-centered use.

Essentials

- Whole-body MRI screening detects cancer in 1–2% of asymptomatic individuals, with incidental findings reported in up to 97%.
- Radiologist-led programs have the advantage of ensuring methodological rigor, standardized reporting, and ethical oversight compared with direct-to-consumer models.
- High incidental finding rates necessitate informed consent, clear patient-focused communication, and evidence-based reporting and management guidelines to reduce unnecessary downstream testing and cost.

## Introduction

Whole-body magnetic resonance imaging (WB-MRI) for early disease detection has garnered increasing attention in the last several years.^1–3^ Initially adopted in select clinical scenarios, such as cancer staging or surveillance in high-risk populations with cancer predisposition,^4^^,^^5^ WB-MRI is now gaining traction for screening normal risk individuals. This is largely driven by for-profit private enterprises, including Ezra and Prenuvo, which have been offering WB-MRI screening to asymptomatic, healthy individuals since 2018. As the demand for personalized preventive care grows and the healthcare diagnostic paradigm shifts from a reactive to a proactive model, the use of screening WB-MRI will likely continue to expand, with or without the scientific rigor of large, randomized control trials demonstrating its value. Despite ongoing skepticism within the broader medical community,^6^ radiologists have an opportunity to thoughtfully engage with screening WB-MRI and help shape its development, rather than dismiss it in its early stage. Radiologist-led implementation of screening WB-MRI within academic and private practices offers the opportunity to ensure methodological rigor and ethical oversight, which may not be inherent in existing direct-to-consumer models. However, integrating screening WB-MRI into routine radiologic practice poses distinct logistical challenges. This review outlines the current evidence and evolving landscape of screening WB-MRI, describes the development and implementation of a program within an academic radiology practice, and discusses the downstream implications and cost-effectiveness of WB-MRI screening.

## Current landscape of screening WB-MRI

Historically, medical guidelines have recommended whole body MRI for diagnostic imaging in select oncologic populations, radiosensitive populations, and children. For example, guidelines from the European Society for Medical Oncology and the American Society of Clinical Oncology advocate the use of WB-MRI for staging prostate cancer when conventional imaging is negative or equivocal, due to its superior detection of metastases compared to CT or PET/CT.^7^^,^^8^ The International Myeloma Working Group (IMWG) guidelines recommend WB-MRI as the initial imaging technique for patients with possible solitary plasmacytoma, due to its superior diagnostic performance over CT for osseous lesion detection.^9^ WB-MRI is also recommended for screening in patients with cancer predisposition syndromes including Li-Fraumeni syndrome (LFS), hereditary paraganglioma and pheochromocytoma syndromes, constitutional mismatch repair deficiency syndrome, and hereditary retinoblastoma.^10^ Studies in such populations predisposed to higher cancer risk have demonstrated high diagnostic cancer yields of 5–10%;^11^^,^^12^ therefore, in these populations, WB-MRI is considered warranted and generally accepted by the medical community.

In contrast, the recent interest and use of WB-MRI for screening asymptomatic low risk individuals have generally been met with caution and considerable skepticism in the medical community.^13^ Screening examinations aim to identify disease at an early, more treatable stage in asymptomatic individuals. However, for a screening test to offer true clinical value, it must be applied to a population with a sufficiently high pretest probability of the disease. This is because screening low-prevalence populations can yield a large proportion of false positives, even with highly specific tests.^14^ There are multiple forms of screening offered to the general population, such as mammography, colonoscopy, and Papanicolaou (Pap) test; however, whole-body screening MRI for the general population is not currently endorsed by most medical professionals as its benefits to long-term patient outcomes are unclear.

Due to the lack of trial data demonstrating utility of screening WB-MRI, The American College of Radiology (ACR) issued a statement in 2023 stating that it does not believe there is sufficient evidence to justify recommending total-body screening for patients without clinical symptoms, risk factors, or a family history suggesting underlying disease or serious injury.^6^ The limited data currently available are largely retrospective and focused on the diagnostic yield of clinically significant findings on screening WB-MRI, without accompanying long term outcome data.^3^^,^^15^ Such studies should be interpreted with caution. A frequently cited cautionary tale is the thyroid ultrasound screening program in asymptomatic individuals in Korea in the early 2000s, which led to a 15-fold increase in the diagnosis of thyroid cancer, without a decrease in thyroid cancer mortality, due to the detection of clinically insignificant thyroid cancers.^16^ This led to many unnecessary surgeries with associated morbidity, highlighting the negative potential of screening overdiagnosis.

### Current evidence

Three systematic reviews/meta-analyses regarding screening WB-MRI have been published to date. The most recent systematic review by Martins da Fonseca et al. included 10 studies (all published after 2015), with 2 of the studies including intravenous contrast, comprising 9,024 asymptomatic individuals, with a reported biopsy rate of 2–22% and a pooled confirmed cancer detection rate of 1.57% (95% CI 1.22–2.03). The study demonstrated low but significant heterogeneity (I2 = 31.3%).^17^ A systematic review from 2020 by Zugni et al. that focused on cancer screening included 12 studies from as far back as 2005, comprising > 6000 screening WB-MRI examinations in asymptomatic patients, demonstrating 17,961 abnormal findings, 91% of which were benign, 9% which required further investigations, and 0.5% of which were suspicious for cancer, with an overall histologically confirmed cancer rate of 1.1%.^3^ Three of the studies in this systematic review included WB-MRI screening with IV contrast and cardiac MRI sequences, and one study included a whole-body MR angiogram. A systematic review from 2019 by Kwee et al. that included 12 studies beginning as early as 2005 (4 with cardiac MRI and MRA of large extracranial arteries, 2 studies including MR colonography, and 1 including breast MRI), comprising > 5000 asymptomatic individuals demonstrated pooled prevalences of critical and indeterminant findings of 13.4% and 13.9%, respectively. The prevalence of these findings was higher in studies that included cardiovascular and/or colon MRI in the protocol. False positive rates were reported in 6 of the studies with pooled proportions of 16%.^15^

Recently, the Hercules and Polaris trial run by Prenuvo is expected to enroll up to 10,000 mostly asymptomatic individuals undergoing screening non-contrast WB-MRI over a 10-year period. Early results presented at ASCO 2025 reported that among 1,011 asymptomatic participants, 93% had at least one previously undiagnosed finding, with 29% of those findings requiring follow-up or treatment. Of those that underwent biopsy, 50% of biopsies were confirmed as cancer, with a confirmed cancer detection rate of 2.2%.^18^

## Implementation of a screening WB-MRI program

### Protocol

**Figure 1:**
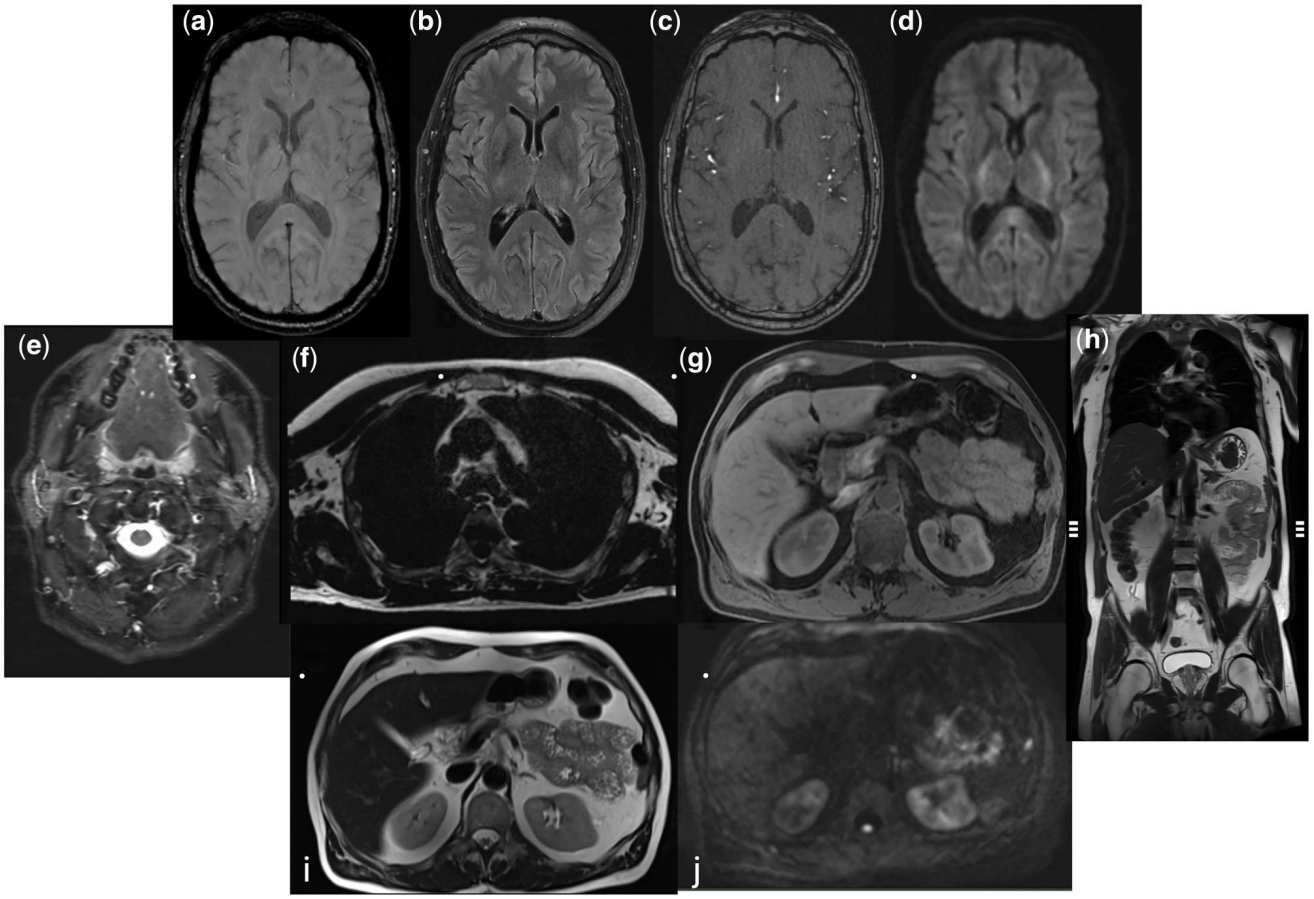
Representative MR Sequences of the Screening Whole Body MRI Protocol. Axial Brain Susceptibility Weighted Imaging (a), Fluid Attenuated Inversion Recovering (FLAIR) (b), Time of Flight MR Angiogram (c), High b-value (b=800) diffusion weighted imaging (d), axial Short Taue Inversion Recovery (STIR) through the neck (e), axial T1-weighted isotropic gradient recalled echo (GRE) with Dixon through the chest (f), whole body axial T1-weighted GRE with Dixon (g), coronal T2-weighted Half Fourier Single Shot Turbo Spin Echo (HASTE) through the abdomen and pelvis (h), and axial whole body T2-weighted HASTE (i), and diffusion weighted imaging (b=800) (j).

Generally, a screening WB-MRI protocol is performed without IV contrast on either a 1.5 T or 3 T scanner, and includes axial T1-weighted, T2-weighted, and diffusion weighted (DWI) sequences from vertex to mid-thigh.^19^ At our institution, the T1-weighted sequences are gradient echo (GRE) Dixon acquisitions, which produce in-phase, out-of-phase, fat, and water only images, and may be acquired isotopically in the chest to obtain sagittal and coronal reconstructions. Axial T2-weighted images are acquired using single-shot or half-acquisition turbo spin echo (HASTE) sequences without fat-suppression from skull base to thigh. For brain imaging, we obtain axial brain T2 fluid attenuation inversion recovery (FLAIR), axial 3D time of flight (TOF) images through the head for aneurysm detection, and short tau inversion recovery (STIR) sequence through the neck. Diffusion-weighted echo-planar imaging is obtained through head, neck, chest, abdomen, and pelvis, with at least two b-values to calculate the corresponding apparent diffusion coefficient (ADC) map (with the highest b value ranging between 800 and 1000 s/mm^2^ and the lowest not lower than 50 s/mm^2)^. In our protocol, we use free-breathing DWI for whole body MRI applications, in which simultaneous multi-slice (SMS) acquisition is used to accelerate imaging time (by up to 25%) and allow for improved signal to noise ratio compared to conventional single shot breath-hold DWI.^20^ A sagittal small field of view T2-weighted sequence through the pelvis for females is also obtained. The scan time for our WB-MRI screening protocol is 45–55 minutes, and all sequences used are commercially available through major vendors. The detailed protocol is outlined in Table 1 and representative images from our WB-MRI are shown in Figure 1.

**Table 1:** Screening whole body MRI protocol*.

Sequence	ST (mm)	Notes	Coverage
Brain: Axial 3D Susceptibility Weighted Imaging (SWI)	3		Brain
Brain: Axial 3D Time of Flight (TOF) MR Angiogram	0.5		Brain
Brain: Axial T2-weighted Imaging with Fluid Attenuated Inversion Recover (FLAIR) technique	5		Brain
Neck: Axial Short Tau Inversion Recovery (STIR)	5		Neck
A/P: Coronal T2-weighted Half Fourier Single Shot Turbo Spin Echo (HASTE)	5		Abdomen and Pelvis
Chest: Axial T1-weighted Gradient Recalled Echo (GRE) with Dixon Technique, Isotropic	1.5		Chest
Whole Body: Axial T1-weighted GRE with Dixon Technique	5	Fat and water reconstructions	Skull base to mid-thigh
Whole Body: Axial T2-weighted Half Fourier Single Shot Turbo Spin Echo (HASTE)	5		Skull base to mid-thigh
Whole Body: Axial Diffusion Weighted (b = 50–100 sec/mm^2^ and 800–1000 sec/mm^2^)	5	Corresponding ADC map with monoexponetial data fitting	Skull base to mid-thigh
Pelvis: Sagittal T2-weighted Turbo Spin without fat suppression	5	Only performed in females	Pelvis

ST= slice thickness.

* Screening Whole Body MRI can be performed on 1.5 T or 3 T scanners.

### Equipment

Designing a WB-MRI screening protocol requires balancing comprehensive diagnostic coverage with efficient scan time to ensure feasibility and patient comfort. The introduction of compressed sensing in the 2000s enabled reconstruction of high-quality images from undersampled data, markedly shortening acquisition time.^21^ More recently, deep learning–based reconstruction algorithms, such as Siemens *Deep Resolve*, GE *AIR Recon DL*, and Philips *SmartSpeed,* which are commercially available, have further enhanced signal-to-noise ratio, spatial resolution, and artifact suppression, allowing up to three-fold faster scans without compromising image quality,^22^^,^^23^ although these algorithms are not required for WB-MRI. Whole-body coverage requires multiple receiver coils, typically combining head-neck, spine, and phased-array surface coils. High-channel-count coils (≥48 channels) and emerging modular coil arrays (48–128 channels) improve parallel imaging performance and support advanced AI-based reconstructions,^24^ but are not a requirement. For table motion, most MRI systems employ step-and-shoot station acquisition, although newer platforms support continuous table movement, producing seamless whole-body volumes and further reducing total scan time.^25^ Together, these hardware and software innovations have helped transform WB-MRI from a lengthy, research-grade examination into a clinically practical screening tool.

### The role of intravenous contrast in screening Whole-Body MRI

Currently, most institutions perform screening WB-MRI without intravenous contrast administration. Given the uncertain long-term clinical implications of gadolinium deposition in the brain, bones, and solid organs,^26^ as well as the potential for allergic reactions,^27^ the risk of administering gadolinium in an asymptomatic screening population could outweigh the benefits. A prior study involved 2500 asymptomatic individuals who underwent whole-body MRI screening, with 619 and 544 of these patients also undergoing contrast-enhanced MR head angiography and contrast-enhanced breast MRI, respectively. The majority (85%) of findings warranting follow-up or intervention in these patients were identified on non-contrast WB-MRI, with only 15% detected exclusively on contrast-enhanced sequences, most of which were BI-RADS ≥ 3 lesions on contrast enhanced breast MRI.^28^ However, due to the absence of a pathological or clinical reference standard in this study, it remains unclear whether these additional findings detected with intravenous contrast represented true or false positives. Further research is needed with a head-to-head comparison to determine the incremental diagnostic value of intravenous contrast use for screening WB-MRI.

### Body composition metrics

**Figure 2:**
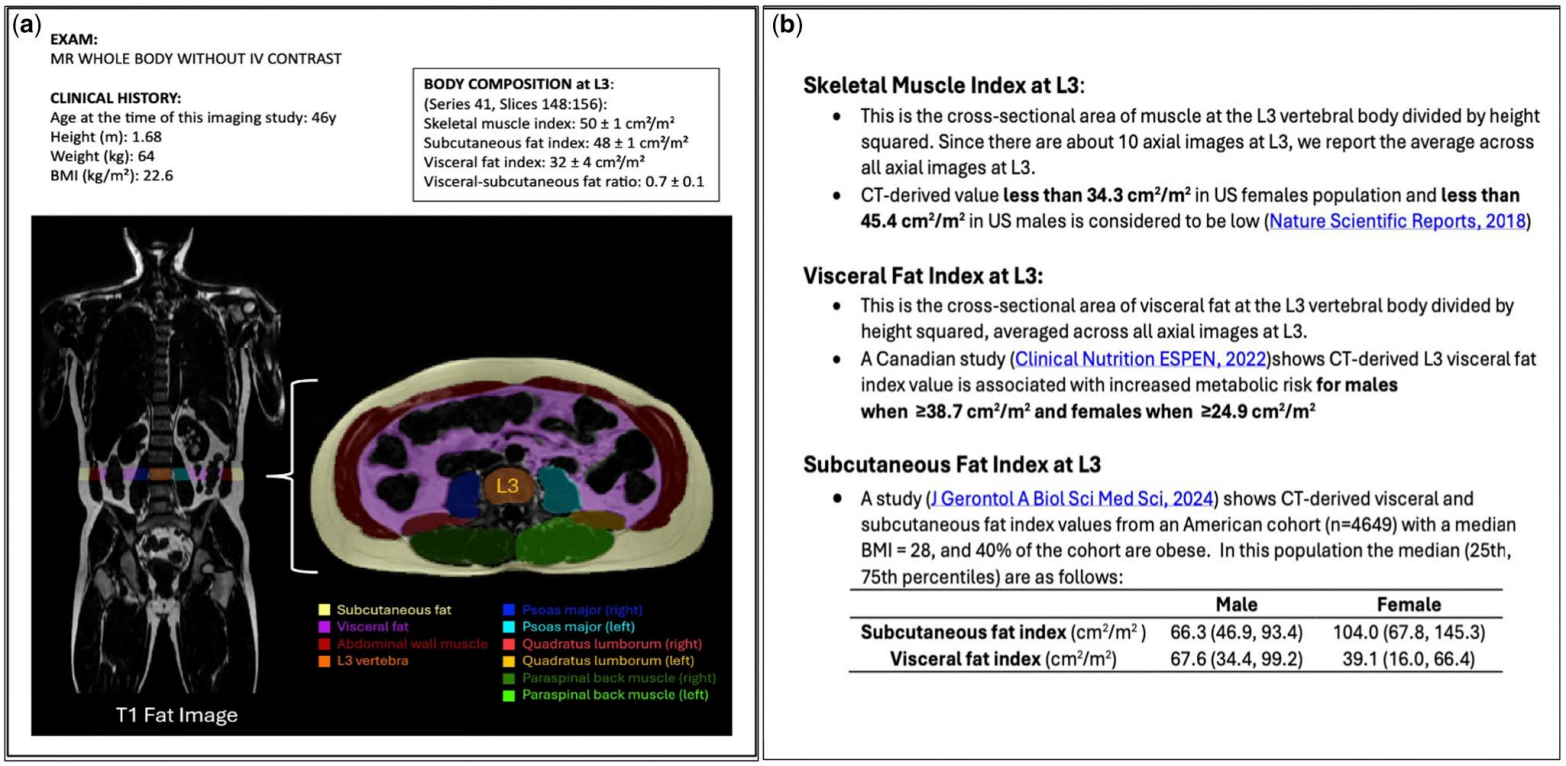
A report of body composition metrics performed on our screening WB-MRI using our proprietary AI deep learning model which automatically segments subcutaneous fat, visceral fat, and skeletal muscle at the L3 vertebral body (a) and provides population-based reference standards for these measurements (b).

In addition to its role in cancer detection, WB-MRI screening can provide body composition metrics to enhance risk stratification. Recent studies have highlighted the limitations of body mass index (BMI) as a sole measure for assessing obesity-related risks^29^ and large imaging-based studies have demonstrated that increased visceral fat, regardless of BMI, is significantly associated with elevated risks of cardiovascular disease and type 2 diabetes.^29^ In our in-house program, we use a proprietary artificial intelligence (AI) deep learning model to automatically segment subcutaneous fat, visceral fat, and muscle mass at the L3 vertebral body level, which is compared to population-based reference values drawn from the literature (Figure 2). The L3 vertebral body level was selected because cross-sectional muscle and fat measurements at this level strongly correlate with whole-body body composition and are widely validated in prior studies.^30^^,^^31^ In our in-house program of model-assisted segmentation, a proprietary 3D nnU-net based artificial intelligence (AI) deep learning model automatically segments abdominal subcutaneous fat, visceral fat, and muscle through a pipeline where every WB-MRI is imported with fat/muscle labels into ITK-snap (a free open-source segmentation platform). Labels on all slices (about 10) at the L3 level are manually reviewed by a radiologist and corrected as necessary to calculate fat and skeletal muscle indices for comparison to population-based reference values drawn from the literature. Alternatively, body composition analysis can be performed using AMRA Profiler (AMRA Medical), a commercially available, FDA-cleared software platform that provides automated, standardized quantification of visceral fat, subcutaneous fat, and skeletal muscle from MRI and has been validated across multicenter cohorts.^32^ Quantification of body composition may allow for a more nuanced analysis of overall patient risk.

### Comparison studies

The importance of reviewing a patient’s medical records and available prior imaging studies cannot be overstated when interpreting screening WB-MRI.^28^ Compared with stand-alone WB-MRI centers, radiologist-led screening programs within established practices often benefit from access to patients’ prior imaging and electronic medical records, allowing for better informed interpretations. A prior study that evaluated over 1,000 abdominal CT and MRI reports found that 4.1% of recommendations for additional imaging or intervention could have been avoided through review of prior imaging, more than half of which involved studies from different modalities and nearly one-third from different body regions.^33^ Although no published studies have yet assessed the value of prior imaging review in WB-MRI screening, it is likely that the benefits are even greater in this setting, where incidental findings are common.

### Reporting: Screening WB-MRI lexicon

A standardized reporting lexicon is essential for WB-MRI screening. Currently, ONCO-RADS is the only established lexicon, developed specifically for WB-MRI in patients with cancer predisposition syndromes.^28^ This system assigns a category to each anatomical region (bones, head, neck, chest, abdomen, pelvis, and limbs), ranging from: ONCO-RADS 1 (normal finding), ONCO-RADS 2 (benign finding, highly likely), ONCO-RADS 3 (benign finding, likely), ONCO-RADS 4 (malignant finding, likely), to ONCO-RADS 5 (malignant finding, highly likely). However, ONCO-RADS does not incorporate clinically significant incidental findings unrelated to cancer, such as aortic aneurysms, which are instead reported in free text under the “other findings” section, and therefore, we do not use ONCO-RADS at our institution for screening WB-MRI. Given that most findings in screening WB-MRI are incidental and non-oncologic in nature, there is a clear need for a dedicated standardized lexicon tailored to this population. In our current reporting system, we categorize findings using a simplified structure:^1^ no follow-up required,^2^ follow-up required (non-critical), or^3^ follow-up required (critical). As the use of screening WB-MRI continues to expand, the development of a standardized lexicon, ideally through a multidisciplinary expert consensus, is critical to ensure consistent interpretation and communication of results. The screening WB-MRI template used at our institution is outlined in Table 2.

**Table 2:** The structured radiologist reporting template used at our institution (with pertinent negatives) for screening whole-body MRI (WB-MRI).

MR WHOLE BODY WITHOUT IV CONTRAST
CLINICAL HISTORY: Screening
TECHNIQUE:
PRIOR IMAGING:
FINDINGS:
**BRAIN**
Parenchyma: Normal brain volume. No acute infarct or acute intracranial hemorrhage. No parenchymal signal abnormality. No evidence of mass lesion.
Ventricles and Extra-Axial Spaces: No hydrocephalus. No extra-axial collection.
Sinuses: The paranasal sinuses and mastoid air cells are clear.
Vessels: No evidence of proximal intracranial arterial aneurysm. No hemodynamically significant stenosis of the proximal intracranial arterial vasculature.
**NECK**
Nasopharynx: No significant abnormality.
Suprahyoid Neck: No significant abnormality in the oropharynx, oral cavity, and bilateral salivary gland tissues.
Infrahyoid Neck: No significant abnormality in the hypopharynx, larynx, and proximal subglottic airway.
Thyroid: Unremarkable.
Lymph Nodes: No adenopathy.
**SPINE**
Alignment: Normal.
Vertebral Bodies: Unremarkable.
Spinal Cord: Normal.
Degenerative Changes: No degenerative changes
**CHEST**
Lungs: No nodule greater than 4 mm. Please note that the parenchyma is not well evaluated with MR.
Heart and Vasculature: The heart is normal in size. No pericardial effusion. The aorta and main pulmonary artery are normal in caliber.
Pleura: No pleural effusion.
Lymph Nodes: No mediastinal, hilar, or axillary lymphadenopathy.
**ABDOMEN AND PELVIS**
Liver: Smooth in contour. No segmental atrophy or hypertrophy. No signal loss on opposed phase imaging to suggest steatosis.
Biliary System: No biliary ductal dilatation. No gallstones.
Pancreas: No pancreatic ductal dilatation.
Spleen: Normal in size.
Adrenal Glands: No adrenal nodule.
Kidneys: No hydronephrosis.
Urinary Bladder: Unremarkable
Reproductive Organs: Unremarkable
Lymph Nodes: No abdominopelvic lymphadenopathy.
Peritoneum: No ascites.
Bowel: No bowel dilatation.
Abdominal Aorta: No aneurysm.
Abdominal Wall: No hernia.
Osseous Structures and Soft Tissues: No suspicious lesion.
IMPRESSION:

### Management of findings

Reportable findings are exceedingly common on screening WB-MRI, with reported rates ranging from 78% to 97% of individuals with 10–25% of findings deemed clinically significant or requiring further evaluation.^1–3^ Only a limited number of studies have comprehensively reported the specific types of findings detected in asymptomatic individuals. Across major cohorts, the most common actionable findings (excluding musculoskeletal findings such as disc bulges), included complex renal cysts or solid renal masses (1.7–5.3%), hepatic lesions such as hemangiomas or focal nodular hyperplasia (2.5–11%), complex ovarian cysts or tumors (2.6–6.3%), thyroid nodules (2.2–6.8%), and adrenal adenomas (0.8–1.7%).^2^^,^^28^^,^^34^^,^^35^ Interestingly, and in contrast to our institutional experience, pancreatic cystic lesions were rare (<2% across studies), and intracranial aneurysms were not identified in any of the published WB-MRI screening cohorts. It remains uncertain whether this discrepancy reflects differing reporting practices, where pancreatic cysts smaller than 1 cm may not have been reported in these cohorts, or differences in imaging protocols, particularly for intracranial aneurysms, as our protocol includes 3D noncontrast time-of-flight (TOF) brain imaging.

**Figure 3:**
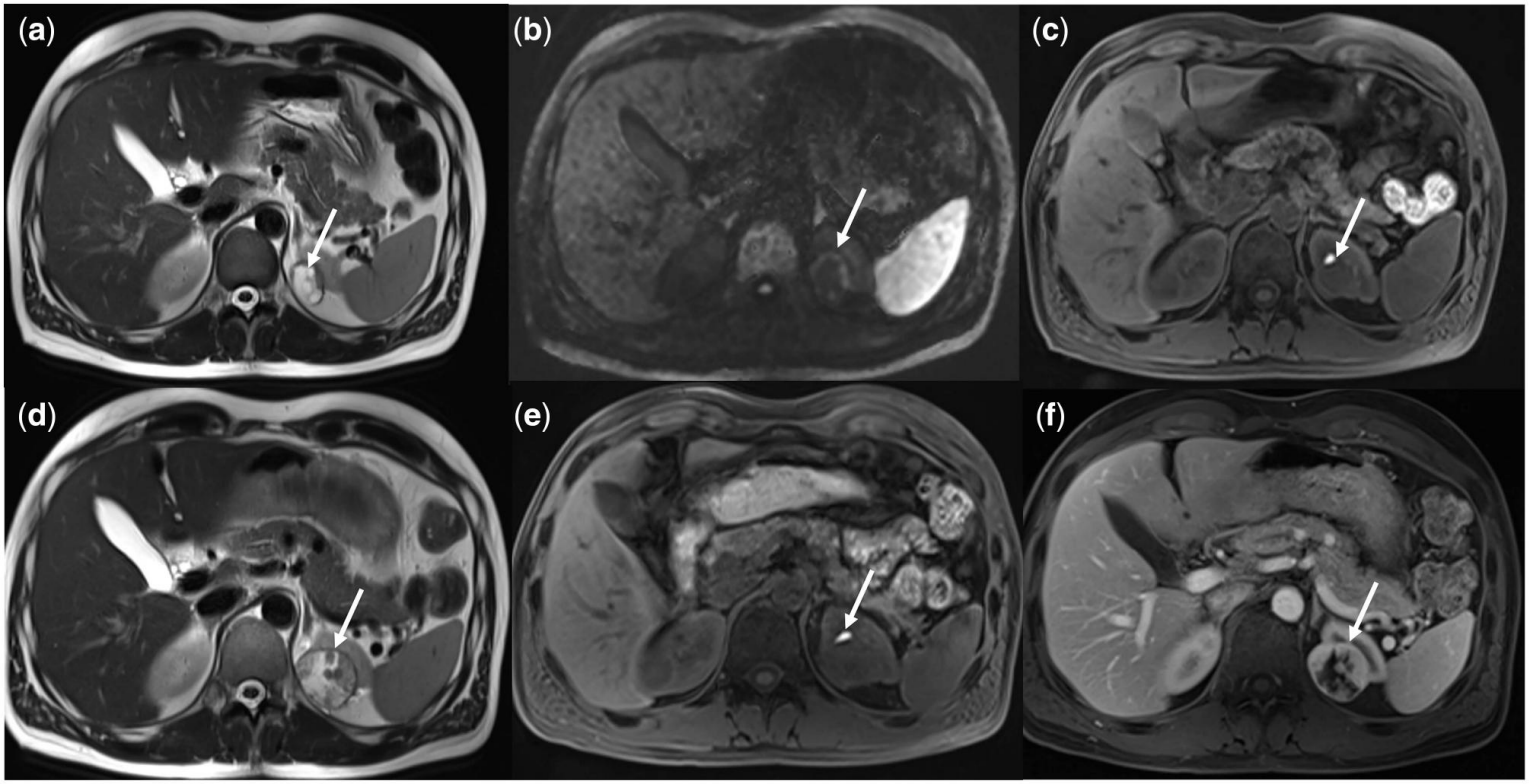
True Positive Finding on Screening WB-MRI. An asymptomatic patient underwent screening whole-body MRI (WB-MRI). Initial baseline WB-MRI without IV contrast, including axial T2-weighted single-shot fast spin-echo (SSFSE) sequence (a), diffusion-weighted imaging (calculated b = 1600 mm–2/s) (b), and T1-weighted gradient-recalled echo (GRE) sequence (c), demonstrated a 2.1-cm left upper pole renal lesion (arrows, a–c) that was T2-heterogeneous with scattered foci of T1-hyperintensity and peripheral faint diffusion restriction (ADC not shown). The lesion was interpreted as a hemorrhagic cyst versus a solid mass, and MRI abdomen with and without IV contrast was recommended. The patient deferred immediate follow-up. One year later, the patient underwent regular scheduled annual screening WB-MRI (with IV contrast to assess the renal mass), demonstrating interval growth of the lesion to 3.6 cm with internal enhancement (arrows, d, e, f). Partial nephrectomy confirmed clear cell renal cell carcinoma.

**Figure 4:**
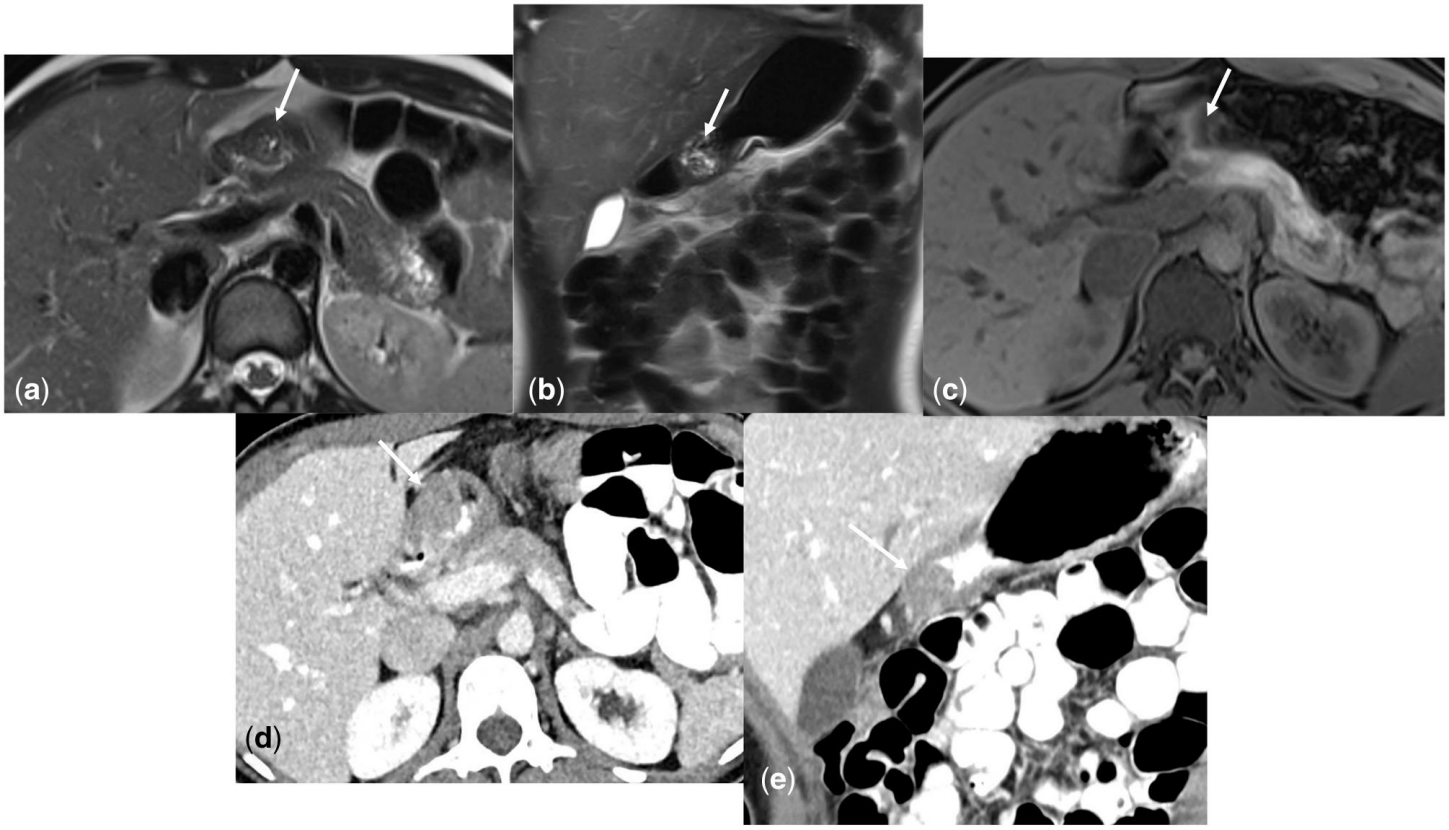
False Positive Finding on Screening WB-MRI. 38-year-old asymptomatic patient underwent screening whole-body MRI (WB-MRI) without IV contrast, including axial and coronal T2-weighted single-shot fast spin-echo (SSFSE) sequences (a, b) and T1-weighted gradient-recalled echo (GRE) sequence (c), which demonstrated a 1.4 cm T2 isointense lesion in the gastric antrum with internal foci of T2-hyperintensity (arrows a and b). The lesion was poorly visualized on the T1-weighted sequence (arrow, c) due to artifact and was not visualized on diffusion weighted imaging (not shown). As this was poorly visualized on MRI due to artifact from adjacent bowel gas, a contrast enhanced abdomen CT was recommended, redemonstrating the antral mass on axial and coronal CT (arrows d and e). This was interpreted as a gastric mass, possibly a gastrointestinal stromal tumor. The patient underwent upper endoscopy and biopsy with results yielding ectopic pancreatic tissue.

**Figure 5:**
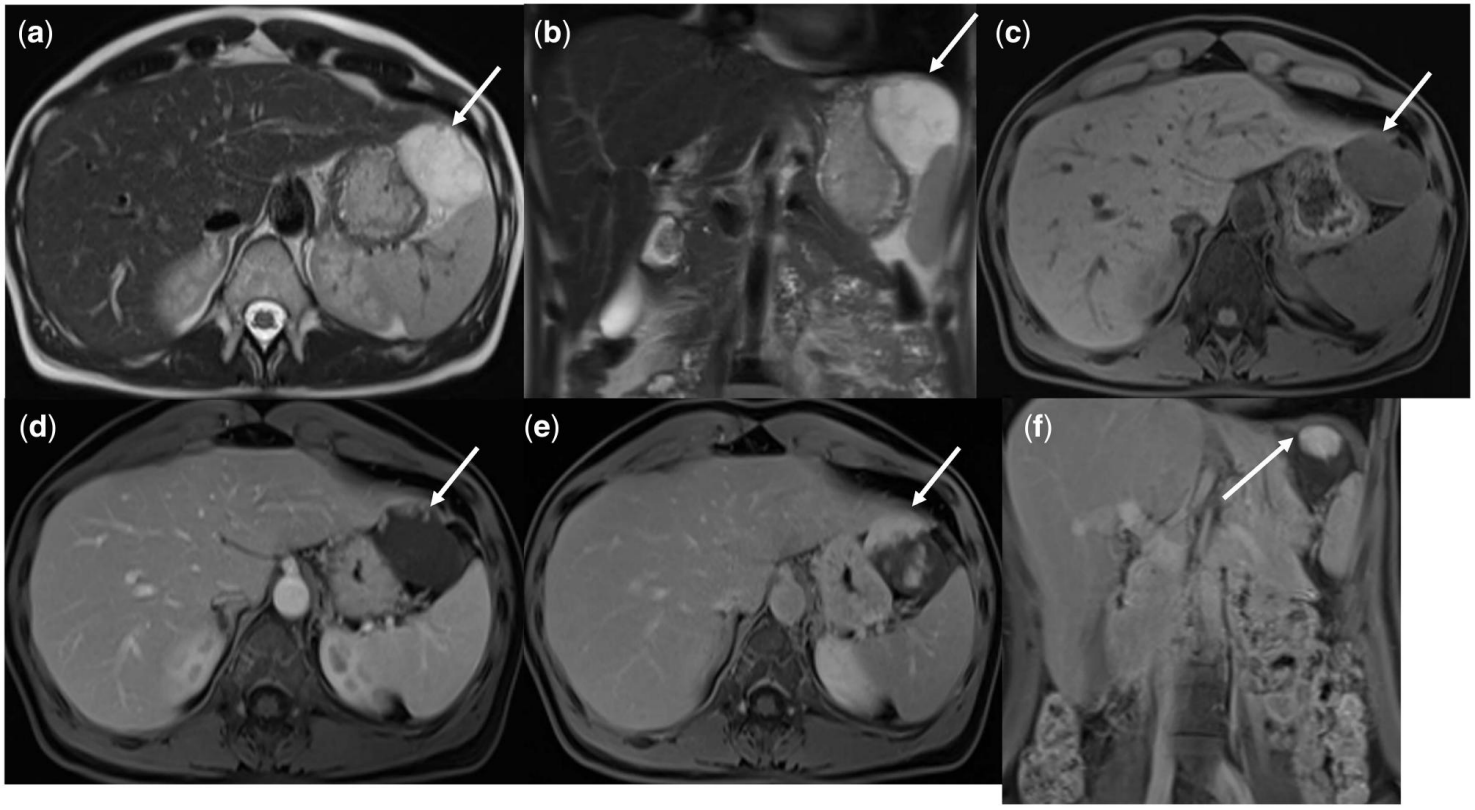
False Positive Finding on Screening WB-MRI. 54-year-old asymptomatic patient underwent screening whole-body MRI (WB-MRI) without IV contrast, including axial and coronal T2-weighted single-shot fast spin-echo (SSFSE) sequences (a, b) and T1-weighted gradient-recalled echo (GRE) sequence (c), which demonstrated a 4.2 cm T2 hyperintense mass in the left upper quadrant (arrows a, b, c), which was read as indeterminant and possibly representing a gastric mass, and a contrast enhanced abdomen MRI was recommended. Contrast enhanced MRI performed 7 days later with axial T1-weighted GRE during the late arterial (d) and portal venous (e) phases as well as coronal T1-weighted GRE during the portal venous phase (f) demonstrated that the left upper quadrant mass demonstrated peripheral nodular discontinuous enhancement progressing centripetally from late arterial to portal venous phase images (arrows, d, e, f), representing an exophytic segment II hepatic hemangioma.

Evidence-based management guidelines should be incorporated into WB-MRI reporting whenever possible to ensure appropriate follow-up recommendations. However, many existing guidelines were developed for other imaging modalities and do not directly apply to noncontrast WB-MRI. For example, thyroid nodules are typically evaluated by ultrasound-based criteria;^36^ complex renal cysts are classified using the Bosniak v2019 system,^37^ pancreatic cysts are managed with either the Kyoto or American College of Radiology guidelines,^38^^,^^39^ and ovarian cysts are usually managed according to O-RADS,^40^ all of which include criteria on contrast enhanced imaging. While we use established management guidelines for findings at our institution (Table 3), the criteria for identifying solid tissue or mural nodules in a cystic lesion were adapted for non-contrast WB-MRI, with any T2-iso or hypointense nodular appearing component (without marked T1-hyperintensity), interpreted as potentially solid and requiring contrast MRI for confirmation. Markedly T1-hyperintense lesions are managed in accordance with established guidelines: a T1-hyperintense/T2-hypointense ovarian lesion is classified as a likely endometrioma; a homogeneously T1-hyperintense renal lesion is categorized as Bosniak II; and a heterogeneously T1-hyperintense renal lesion warrants further evaluation with contrast-enhanced MRI^34^ (Figure 3). As WB-MRI becomes more widely adopted, the development of standardized, evidence-based recommendations tailored to noncontrast MRI protocols will be essential to optimize clinical utility while minimizing unnecessary follow-ups. However, findings that fall outside existing guideline frameworks remain a challenge for WB-MRI and may lead to unnecessary downstream testing or intervention (Figures 4 and 5).

**Table 3:** Management of Common Incidental Findings on Screening WB-MRI.

Incidental Finding	Incidence on WB-MRI	Recommendation
Complex renal cyst or presumed solid renal mass (non T2 hyper-/non-markedly T1 hyper-intense)	1.7–5.3%	Complex Renal Cyst: Apply Bosniak v2019* • If Bosniak IIF: follow-up MRI C+ in 6 mo. • If Bosniak III or IV: immediate follow-up MRI C+ Presumed Solid Renal Mass (non T2 hyper-/non-markedly T1 hyper-intense: • MRI C+^37^
Non-cystic hepatic lesion	2.5–11%	• If fluid T2-hyperintense (either cyst or suspected hemangioma): No follow-up • If non-T2-hyperintense: MRI C+
Ovarian cyst	2.6–6.3%	Apply ACR O-RADS*^40^
Thyroid nodule	2.2–6.8%	Thyroid US^36^
Adrenal nodule	0.8–1.7%	Apply ACR guidelines^59^
Pancreatic cyst	≤ 2%	Apply Kyoto Guidelines*^38^
Intracranial aneurysm	Not reported	Refer to neurology or neurosurgery
Pineal Cyst	1–25%	Apply ACR guidelines^60^
Pituitary Microadenoma	10–30%	Refer to endocrine for baseline endocrine evaluation^60^.
Brain unidentified Bright Objects (focal areas of brain signal hyperintensity)	50% of those in 5th decade of life	No imaging follow-up needed in asymptomatic patients^61^
Degenerative spine changes or disc related spinal stenosis (neuroforaminal or central)	50–60% of those in 5th decade of life	No imaging follow-up needed in asymptomatic patients^62^^,^^63^
Osseous Lesions	2–10%	Apply ACR Bone-RADS^64^

ACR = American College of Radiology; O-RADS = Ovarian-Adnexal Reporting and Data System; US = ultrasound; MRI = magnetic resonance imaging; C+ = contrast-enhanced; WB-MRI = whole-body magnetic resonance imaging.

* These systems are designed for contrast-enhanced imaging and include recommendations based on the presence of a solid nodule or component. We consider any mural nodule or component that is T2-isointense or T2-hypointense relative to fluid (and not markedly T1-hyperintense) as a possible “enhancing” or solid element, with a recommendation of contrast enhanced MRI given for confirmation.

### Communication of results

One of the essential components of a successful WB-MRI screening program is the appropriate and responsible communication of imaging results. Patients who have undergone screening WB-MRI have reported on social media that they were left to interpret complex imaging reports on their own, leading to unnecessary panic and distress. In one widely shared account, a patient who underwent a screening WB-MR received her radiology report via an online portal at 8:30 p.m., but her scheduled consultation with a nurse practitioner was not until 24 to 48 hours later, and she was not supplied with any contact number to call in the interim. The report noted a “splenic artery aneurysm,” a term she was unfamiliar with, and a quick online search led her to believe she had a 10% chance of imminent rupture and death. With no one available to speak to, she drove to the emergency room in a panic.^41^ This example underscores the need for timely, comprehensible, and compassionate communication of actionable findings, delivered directly to the patient in clear, jargon-free language, along with a straightforward explanation of next steps.

**Figure 6:**
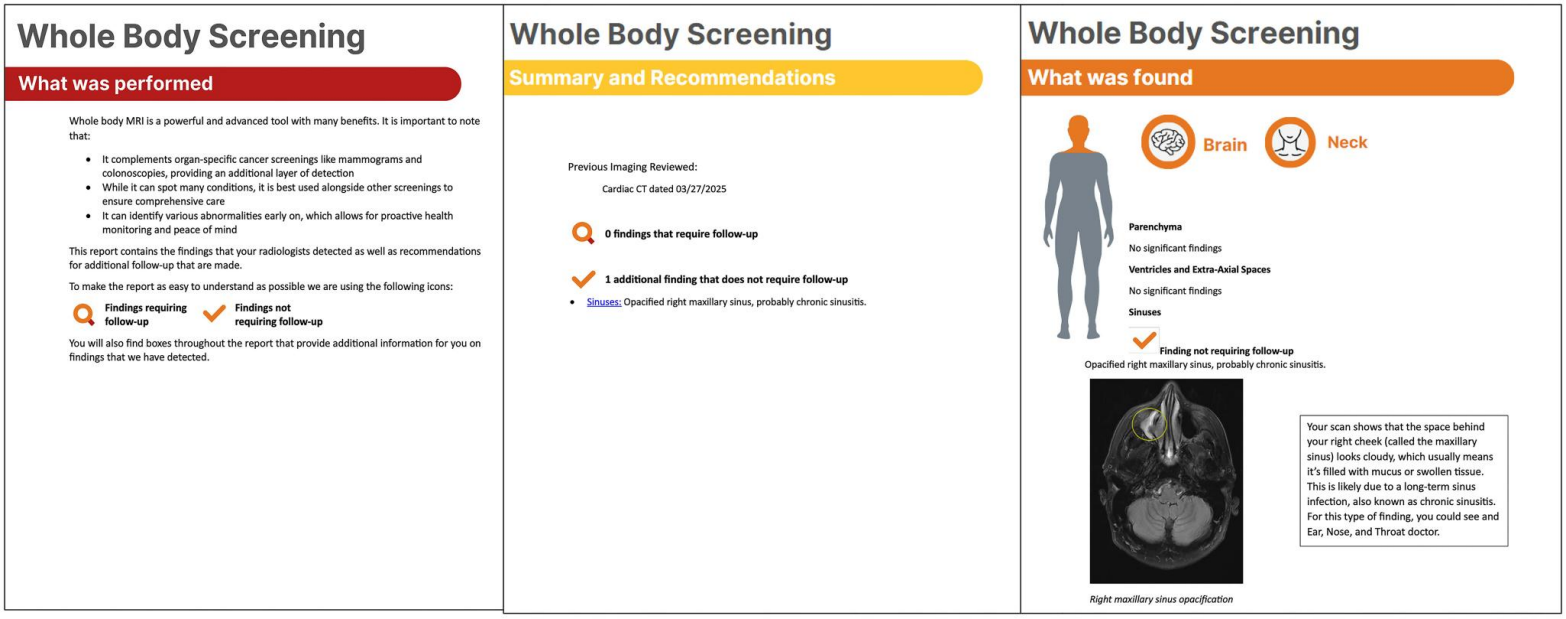
Example of Portions of the Screening WB-MRI “Patient-Centered” Report at our Institution. The original radiology report is translated into a patient-friendly version that is free of medical jargon, using a large language model artificial intelligence program, which is then manually checked for accuracy by our radiology nurse practitioner. The patient centered version explains the examination including every anatomic area imaged and outlines the findings requiring follow-up and those not requiring follow-up in each anatomic region, along with a representative image of every finding detected.

WB-MRI screening programs should consider issuing a full clinical report and a “patient-centered” version for each examination, the latter designed to eliminate medical jargon and enhance accessibility. In our program, we developed a comprehensive library of Patient-Friendly Explanations (PFx) for the most common findings using a large language model (LLM)-assisted workflow. We have curated each PFx to convey clinically accurate information in plain language. The radiologist identifies findings in the preliminary report using a macro that includes a short descriptor and an image number from PACS. A secondary LLM matches the finding to the most appropriate PFx from the database, and the script also automatically extracts key images from PACS, creating a draft report that pairs each finding with a representative image and corresponding PFx. The PFx templates were validated using an agentic workflow that included iterative readability and accuracy checks, with all outputs reviewed by a radiologist agent prior to acceptance, ensuring quality control of the generated explanations. All LLM processing is performed in-house using commercial models on de-identified inputs that contain no protected health information.^42^ The patient-centered report is organized by anatomical region (brain, neck, chest, abdomen/pelvis), with findings summarized on the first page and categorized by whether follow-up is recommended (Figure 6). Based on prior research indicating that patients prefer to view their imaging,^43^ a single representative MR image, with annotation, is included for each finding, regardless of clinical significance. Draft reports are reviewed for accuracy by a radiology nurse practitioner and radiologist prior to finalization. In cases without actionable findings, the patient-centered report is sent to the patient and referring physician via the patient portal during business hours and includes the name and contact information of the nurse practitioner and interpreting radiologists, offering patients the opportunity to initiate direct communication if desired. For patients without a referring physician, the radiology nurse practitioner contacts the patient to discuss results in addition to sending the report.

### Referral base and marketing

Engaging primary care physicians (PCPs) is a critical component in the successful implementation of a screening WB-MRI program. As the first point of contact for most patients, PCPs are uniquely positioned to identify individuals who would be interested in WB-MRI screening and to counsel them on their potential advantages and limitations. Conducting educational meetings with referring primary care physicians to review the logistics and clinical considerations of a screening WB-MRI program may be helpful. Discussions may include associated out-of-pocket costs, review of common findings, potential for false positive findings leading to unnecessary follow-up, and limitations of WB-MRI for certain cancers including colon cancer and lung nodules. To reduce the administrative burden on PCPs resulting from this program at our institution, our radiology department assumes responsibility for coordinating referrals for any actionable findings, should the PCP prefer. In such cases, the nurse practitioner consults with the interpreting radiologist to determine the need for referral. For example, for an incidental pancreatic cyst we determine whether to refer the patient to gastroenterology based on established guidelines and arrange guideline-concordant follow-up imaging if needed.^38^

### Reimbursement

Reimbursement for screening WB-MRI remains a significant barrier to widespread adoption, as most commercial and government insurance plans do not currently cover this exam due to the absence of evidence linking screening WB-MRI to survival benefits. However, in select high-risk populations, such as individuals with known cancer predisposition syndromes, WB-MRI may be reimbursed when performed for surveillance, as part of established clinical protocols. Institutions offering WB-MRI outside of these narrow indications often use the unlisted MRI procedure code (CPT 76498) for billing, which does not guarantee reimbursement and usually requires individualized justification. As a result, most screening WB-MRI exams in the general population are currently paid for out of pocket by patients, with costs in 2025 ranging from $500 to $3000. In our program, all our patients for screening WB-MRI currently pay entirely out of pocket.

## Cost effectiveness

A central challenge to implementing whole-body MRI (WB-MRI) screening in asymptomatic individuals is cost-effectiveness. A significant portion of the economic burden is the downstream medical utilization from findings that often ultimately prove benign. In a German population-based cohort of 5019 individuals, 2969 underwent WB-MRI, and 10% of scans yielded findings that prompted further evaluation, resulting in 11.6% higher two-year outpatient costs among those screened (mean €2839 vs €2547), with imaging and specialist consultations as the principal drivers of excess expenditure. Notably, these elevated costs persisted even in participants without reported MRI findings, suggesting a generalized increase in medical surveillance following WB-MRI.^44^ A study examining the cost of incidental findings at CT colonography showed that follow-up of extracolonic findings adds approximately $100–250 per screened individual, even before intervention costs.^45^ A common finding seen on WB-MRI is pancreatic cysts, with approximately 14% prevalence;^46^ the mean downstream cost per cyst is estimated at $460, rising to $872 in cases that lead to further testing.^47^ Adding to the ethical implications is that patients’ insurance currently typically bears the downstream costs of evaluating incidental findings, raising concerns about cost-shifting and resource stewardship, ultimately requiring clearer reimbursement frameworks for screening WB-MRI in the future.

In a threshold cost-effectiveness model, assuming a $1000 cost per WB-MRI in 2025 and an average downstream (“cascade”) cost of $300 per screened individual, we applied a Quality-of-Life Years (QALY) estimate derived from a multicancer early detection (MCED) study from 2024, which found that shifting cancer detection to an earlier stage yielded an average gain of 3.83 QALYs per person with a “shifted” cancer.^48^ Using the standard U.S. willingness-to-pay threshold in 2025 of $100,000 per QALY,^49^ WB-MRI screening would need to achieve an actionable cancer yield of 0.327% (3.27 per 1000 screened) to be considered cost-effective under these assumptions. This estimate is highly dependent upon the proportion of detected cancers that shift stage and improve outcomes, which is an unknown parameter. If only 50% or 25% of detected cancers resulted in such benefit, WB-MRI would require cancer yields of 1.17% (11.74 per 1,000) and 0.63% (6.30 per 1000), respectively, to meet cost-effectiveness thresholds (Figure S1). These projected yields are comparable to or slightly lower than the cancer detection rates currently reported in screening WB-MRI studies (1.1–2.2%). However, the MCED-derived stage-shift rate likely overestimates the benefit achievable with WB-MRI, as cancers identified on noncontract WB-MRI are typically larger, radiographically apparent lesions that may already represent a later stage in disease progression than those detected with blood-based tests.^48^ Robust data on clinical outcomes and QALYs gained per person screened with WB-MRI are essential to enable accurate cost-effectiveness modeling.

## Ethical considerations

### Strategies to mitigate downstream costs

Several strategies may mitigate downstream costs while preserving potential benefits of screening WB-MRI. Structured reporting frameworks, standardized patient disclosure criteria, and multidisciplinary review committees may reduce unnecessary workups for benign findings. Integration of artificial intelligence tools for automated lesion characterization and risk stratification may further enhance specificity. The use of multi-cancer early detection (MCED) assays, blood-based tests that detect circulating tumor DNA or methylation signatures from multiple tumor types, could also assist risk stratification. Recent large-scale evaluations of multicancer early detection (MCED) platforms such as Galleri and Carcimun have shown specificities exceeding 98% and sensitivities of approximately 70–90% for advanced-stage cancers, with lower sensitivities of 25–40% for early-stage disease, while maintaining high specificity (>95%).^50–52^ Integrating MCED assays with WB-MRI may offer a novel, cost-conscious screening model, with MCED results serving as a molecular pre-screen, triaging only individuals with positive or indeterminate blood tests to undergo confirmatory WB-MRI, thereby improving diagnostic yield. Therefore, while universal WB-MRI screening may not be cost effective, one driven by genetically informed assessment of risk may offer a more cost-effective path forward.

### Informed consent

Given the high rate of incidental findings in screening WB-MRI, radiologists have a professional responsibility to ensure that patients are thoroughly informed about the potential for false-positive findings that may prompt additional diagnostic evaluations or interventions. Ethical considerations around informed consent for emerging screening technologies parallel those described in the multi-cancer early detection (MCED) literature.^53^ Similar to MCED frameworks, WB-MRI screening requires transparent disclosure of overdiagnosis risk, the absence of validated screening intervals, and the lack of evidence demonstrating mortality benefit in average-risk populations. Commercial WB-MRI programs such as Prenuvo and Ezra require patients to electronically sign pre-examination acknowledgment forms that outline the high likelihood of incidental findings.^54^^,^^55^ Research screening cohorts, including the German SHIP cohort, similarly provide structured written information and require participants to acknowledge the possibility of incidental abnormalities and follow-up evaluations before imaging in their research informed consent.^28^ At our institution, written consent is not mandated by the IRB; however, every patient receives a dedicated pre-examination counseling call from our radiology nurse practitioner (NP), where they explain what WB-MRI can and cannot detect, review the possibility of incidental or false-positive findings, and discuss the potential need for follow-up imaging or specialist evaluation. The NP also emphasizes that WB-MRI has no proven mortality benefit in average-risk individuals and that optimal screening intervals are unknown. Lastly, similar to pre-test counselling in genetic testing,^56^ patients are made aware that findings identified on screening WB-MRI may have implications for life insurance eligibility, possibly leading to increased premiums, delayed approval, or denial of coverage. If patients have follow-up questions regarding the implications of incidental findings on WB-MRI, they are given the nurse practitioner number and are also informed that the dedicated WB-MRI radiologist is available to speak with them if desired.

### Disparities in access and representation in research

Another significant ethical issue is the inequitable access to WB-MRI screening, which is typically offered as a self-pay service with high out-of-pocket costs, usually costing between $500-$5000. This model effectively limits participation to affluent, health-literate individuals, thereby excluding patients from lower socioeconomic backgrounds and historically underserved communities. If WB-MRI is proven to improve patient outcomes, WB-MRI screening costs could be mitigated in underserved populations by establishing institutional or government-subsidized screening programs, similar to those supporting mammography or low-dose CT lung screening, to offset direct out-of-pocket costs.^57^ To mitigate geographic limitations in access, mobile MRI units or mobile imaging outreach programs could bring scanners closer to underserved communities. For example, a long-running mobile MRI initiative performed over 279,000 scans across rural and remote sites in Spain over 18 years, demonstrating the feasibility of MRI scanning in remote regions.^58^

## Conclusion

Screening whole-body MRI offers the potential for early disease detection but remains limited by uncertain clinical benefit, high rates of reported findings, and downstream costs. As utilization expands, radiologists must lead its responsible integration through standardized protocols, evidence-based management, and transparent patient communication. Radiologist-led programs can ensure methodological rigor, ethical oversight, and consistency in exam quality, while reducing unnecessary testing through guideline-informed follow-up. However, prospective studies are necessary to determine clinical outcomes, cost-effectiveness, and optimal management of indeterminate findings. Until such evidence emerges, cautious, radiologist-directed implementation should guide the adoption of screening WB-MRI.

## Supplementary Material

umag014_Supplementary_Data
