# Supplementary material for: How to implement a radiologist led whole-body MRI screening program
Source: Radiol Adv. 2026 Mar 10;3(2):umag014. doi: 10.1093/radadv/umag014 (PMC13008331; doi:10.1093/radadv/umag014)
Supplement: umag014_Supplementary_Data [file umag014_supplementary_data.zip › Supplement_Data.zip!/Supplemental_material_revised.pdf]

Figure S1. Cost-Effectiveness Threshold Model for Screening Whole-Body MRI

Model Framework

The analysis estimates the minimum actionable cancer detection rate (**p***, per screened person) required for whole-body MRI (WB-MRI) to achieve cost-effectiveness at a willingness-to-pay threshold of $100,000 per quality-adjusted life year (QALY). The model uses the following equation:

$$p^* = \frac{C_{\text{scan}} + C_{\text{cascade}}}{\lambda \cdot G + S}$$

Variable Definitions

Symbol	Definition	Base-Case Value	Rationale / Source
C_{scan}	Cost of a single WB-MRI screen per individual	$1,000	Approximate academic institutional charge
C_{cascade}	Mean downstream "cascade" cost per screened individual (due to incidental findings and follow-up imaging)	$300	Derived from published estimates of incidental findings (pancreatic cysts $460–$870; pulmonary nodules $393–$940)
λ	Willingness-to-pay threshold	$100,000 per QALY	Standard U.S. benchmark
G	QALYs gained per *detected actionable cancer* (cancer shifted to earlier stage)	3.83 QALYs	From a multi-cancer early detection (MCED) model: average gain per shifted cancer (varying by cancer type)
S	Lifetime cost savings per early-stage cancer detected	$15,000	Approximate treatment cost offset (early vs. advanced stage)

Calculation

$$p^* = \frac{1000 + 300}{100{,}000 \times 3.83 + 15{,}000} = \frac{1300}{398{,}000} = 0.00327$$

Thus, WB-MRI screening would need an actionable cancer detection rate of **0.33% (≈3.3 cancers per 1,000 individuals screened)** to achieve cost-effectiveness at $100,000/QALY under these assumptions.

Sensitivity Analysis (Varying the Proportion of True Stage-Shifted Cancers)

Fraction achieving true stage shift (f)	Effective $G = f \times 3.83$	Required yield per 1,000 screens
1.0 (all actionable cancers yield 3.83 QALYs)	3.83	3.3
0.75	2.87	4.3
0.50	1.92	6.3
0.25	0.96	11.7

Figure Legend

Figure S1.
Threshold model illustrating the relationship between scan cost, downstream cascade costs, and per-cancer QALY gains required for whole-body MRI screening to be cost-effective. Using published MCED estimates of 3.83 QALYs gained per shifted cancer, a $1,000 scan, and $300 mean downstream costs, the model predicts a required actionable cancer yield of 0.33% (3.3 per 1,000 screens) to reach a $100,000/QALY benchmark. Sensitivity analysis shows that if only half of detected cancers achieve meaningful stage shift, the threshold increases to roughly 6 per 1,000.
